# Cross sectional survey of human-bat interaction in Australia: public health implications

**DOI:** 10.1186/1471-2458-14-58

**Published:** 2014-01-21

**Authors:** Beverley J Paterson, Michelle T Butler, Keith Eastwood, Patrick M Cashman, Alison Jones, David N Durrheim

**Affiliations:** 1 Hunter Medical Research Institute, University of Newcastle, Newcastle, Australia; 2 Hunter New England Population Health, Newcastle, Australia; 3 Graduate School of Medicine, University of Wollongong, Wollongong, Australia

## Abstract

**Background:**

Flying foxes (megachiroptera) and insectivorous microbats (microchiroptera) are the known reservoirs for a range of recently emerged, highly pathogenic viruses. In Australia there is public health concern relating to bats’ role as reservoirs of Australian Bat Lyssavirus (ABLV), which has clinical features identical to classical rabies. Three deaths from ABLV have occurred in Australia. A survey was conducted to determine the frequency of bat exposures amongst adults in Australia’s most populous state, New South Wales; explore reasons for handling bats; examine reported practices upon encountering injured or trapped bats or experiencing bat bites or scratches; and investigate knowledge of bat handling warnings.

**Methods:**

A representative sample of 821 New South Wales adults aged 16 years and older were interviewed during May and June 2011, using a computer assisted telephone interview (CATI) method. Frequencies, proportions and statistical differences in proportion were performed. Using an α-value of 0.05 and power of 80%, it was calculated that a sample size of 800 was required to provide statistical significance of +/− 5% for dichotomous variables.

**Results:**

One-hundred-and-twenty-seven (15.5%) respondents indicated that they had previously handled a bat, being 22% (48/218) rural and 13% (78/597) urban respondents (*χ*^2^ = 9.8, p = 0.0018). Twenty one percent of males (63/304) had handled bats compared with 12% (64/517) of females (*χ*^2^ = 10.2, p = 0.0014). Overall, 42.0% (n = 345) of respondents reported having seen or heard a warning about handling bats. If faced with an injured or trapped bat, 25% (206/821) indicated that they would handle the bat, with 17% (36/206) saying that they would use their bare hands. For minor scratches, 14% (117/821) indicated that they would ignore the injury while four respondents would ignore major scratches or bites.

**Conclusions:**

Previous human-bat interactions were relatively common. Bat exposures most frequently occurred with sick or injured bats, which have the highest risk of ABLV. On encountering an injured or sick bat, potentially high risk practices were commonly reported, particularly among rural males. It is important to understand why people still handle bats despite public health warnings to inform future communication strategies.

## Background

Bats (order Chiroptera) are the known host reservoirs for a range of viruses and their role in recent emergent infectious diseases has been firmly established [1,2]. More than 60 viruses have been detected in bat tissue [3,4], with bats implicated as reservoir hosts for highly pathogenic Nipah, Hendra, Lyssa and Ebola viruses. With the exception of lyssavirus infections, these agents do not appear to cause overt bat disease [4,5]. Following the discovery of closely related coronaviruses in European and African bats, bats have been identified as the likely hosts of the recently emerged Middle East Respiratory Syndrome (MERS)-CoV [6-8]. Bats’ role in emergent diseases has led to questions whether increased detection has been the result of increased surveillance or of ecological changes [4].

In Australia, public health concerns relate to bats’ role as reservoirs of Australian Bat Lyssavirus (ABLV) and Hendra virus (HeV). Human infection with ABLV may result in a fatal encephalitic disease following exposure to infectious insectivorous bats (Microchiroptera) or flying foxes (Megachiroptera), usually by bite or scratch. The resultant disease has clinical features identical to classical rabies. There have been three ABLV human deaths in Australia, two adults in 1996 and 1998 [9,10] and, tragically, a third death, in an eight year old Queensland boy, in February 2013 [11].

Serological testing and viral studies have confirmed a wide distribution of infected bats along the entire eastern seaboard, including NSW where the Australian population is most concentrated [12]. Approximately 5% of tested Australian bats have evidence of ABLV infection, however this increases to 20% for sick, injured or orphaned bats, depending on the species [13]. As ABLV may cause overt disease in bats, it is more likely that abnormal bat behaviour (acting aggressively, unable to fly or hanging from low branches in proximity to human dwellings) is associated with ABLV infection. Sick bats are more likely to be involved in human-bat or animal-bat interactions [13].

The WHO Expert Consultation on Rabies (2013) states that any bat exposure/bite is considered a category III exposure and post-exposure prophylaxis (PEP) should always be administered [14]. Rabies vaccine is protective against ABLV and pre exposure vaccination is recommended for wildlife carers [15]. In Australia the general public are advised to contact trained or licensed bat handlers or wildlife organisations if they encounter an injured or trapped bat. It is recommended that people who are not vaccinated against rabies should not handle bats. If exposure due to bite or scratch does occur the wound should be infiltrated with human rabies immunoglobulin and a course of four rabies vaccinations should be initiated [16]. These medications are expensive and have previously been in short supply in Australia.

Queensland surveys indicate that, despite various public awareness campaigns, the number of avoidable exposures in community members requiring post-exposure management with rabies vaccine and rabies immunoglobulin, has not decreased in 15 years [17].

Earlier studies have reported notifications to public health units of potential contacts (scratches or bites) with bats [17,18] but no studies have examined human-bat interactions in the Australian public. Wood et al. (2012) noted that human-bat interactions are insufficiently studied [19]. Our study assessed bat exposures amongst the general public; reported actions after encountering injured or trapped bats, or experiencing bat bites or scratches; and respondents’ knowledge of ABLV warnings.

## Methods

### Study participants and study protocol

A representative sample of New South Wales (NSW) residents aged 16 years and older were interviewed during May and June 2011 using a computer assisted telephone interview (CATI) method. NSW is Australia’s most populous state with a population of 7.2 million in 2010. The sample consisted of people who had been randomly selected in 2010 for a population health survey using an electronic telephone directory and geo-coded to ensure satisfactory NSW representation [Hunter New England Area Health Service: *Good For Kids: good for life. Data Dictionary. Baseline Random Household CATI*, Section 1.3 page 1 (unpublished). Newcastle: Hunter New England Area Health Service; 2010]. This group had indicated their willingness to participate in future surveys. The database (containing names and telephone numbers only) formed the sampling frame.

Experienced health telephone interviewers were trained to ensure a consistent approach. Calls were placed between 09.00 and 20.00, with up to seven call attempts made for each individual. Eligibility criteria required that respondents be: 16 years or older, have provided verbal consent and be able to converse in English.

Second round interviews were conducted in June and July 2013 with respondents who stated that they had previously handled bats in the first survey. Calls were placed by authors BJP, MTB and KE between 09.00 and 20.00, with up to seven call attempts made for each individual.

### Interview procedure

The initial interview explored the following issues: prior exposure to bats, including self-reported ‘handling of bats’; risk behaviours; and demographic data to allow comparison with the NSW population as a whole. Participants were questioned to determine whether they regularly worked with animals, including bats. Questions regarding knowledge of bat warnings were posed after they had answered questions regarding their likely behaviour on encountering a trapped or injured bat. Participants were able to provide multiple responses to a number of questions. Participants were given the opportunity to ask questions at interview conclusion, and were sent additional ABLV information if requested. If participants indicated that they had previously been bitten or scratched by a bat or a flying fox, they were followed-up by health protection staff to ascertain exposure risk and initiate treatment if indicated.

Second round interview questions explored how long ago the bat exposure had occurred; whether there were single or multiple bat exposures; and the respondents’ reasons for handling the bat.

### Statistical methods

Analyses were conducted using SAS 9.2 (SAS Institute, Cary, NC, USA). Frequencies, proportions and statistical differences in proportion were performed. Tests of significance used data weighted (age and gender) to the NSW estimated resident population in June 2009 [20]. Socio-economic status was gauged using the disadvantage index of the Socio-Economic Indexes for Areas (SEIFA) from the Australian Bureau of Statistics [21]. Urban areas were determined using Accessibility/Remoteness Index of Australia (ARIA) [21]. Using an α-value of 0.05 and power of 80%, it was calculated that a sample size of 800 was required to provide statistical significance of +/− 5% for dichotomous variables.

### Ethics

Ethics approval was obtained from the Hunter New England Human Research Ethics Committee (approval number 11/04/20/5.12).

## Results

Fourteen hundred telephone numbers were called and 821 interviews completed. There were 166 refusals; 111 respondents did not meet the eligibility criteria; and 302 were unable to be contacted; a participation rate of 83%. Follow-up interviews were completed with 39% (49/127) of respondents who stated that they had previously handled bats. The median age of respondents was 58 years. The study population was compared with the NSW general population (Table 1). There were more females (63%) than males and the age distribution was older in the study group. Participants living in lower socio economic postal areas were over-represented in the study group (64.3% vs 54.6% in the general population) (data not shown). Sixty-eight respondents indicated that they worked with animals, including two veterinarians, four animal volunteer carers, one bat handler and 47 farmers.

**Table 1:** Age and sex of all survey respondents, respondents who had handled bats, and respondents who had not handled bats compared to the general NSW population

	**NSW**	**All respondents**	P-value**	**Previously handled bats**	**Have not handled bats**	**P-value**
	**%**	**% (n)**		**% (n)**	**% (n)**	
Total	100	100 (821)		15.5 (127)	84.5 (694)	
Gender						
Male	49.1 (2772522)	37.0 (304)	<0.001	49.6 (63)	34.7 (241)	0.0027
Female	50.9 (2864788)	63.0 (517)		50.4 (64)	65.3 (453)	
Age*						
Median age	43 years	58 years		56 years	58 years	
16–20 years	6.7 (378100)	1.7 (14)	<0.001	2.4 (3)	1.6 (11)	0.0362
20–29 years	17.9 (1010437)	5.1 (42)		6.3 (8)	4.9 (34)	
30–49 years	35.4 (1995657)	26.2 (214)		24.4 (31)	26.5 (183)	
50–64 years	22.5 (1267767)	31.8 (260)		37.0 (47)	30.9 (213)	
65+ years	17.5 (985349)	35.1 (287)		29.9 (38)	36.1 (249)	
ARIA#						
Urban	68.7 (3872832)	73.2 (597)	0.005	61.9 (78)	75.3 (519)	0.0265
Rural	31.3 (1764478)	26.8 (218)		38.1 (48)	24.7 (170)	

*4 people refused. #6 missing postcodes.

**The p-value is calculated relative to the general NSW population.

#Accessibility/Remoteness Index of Australia.

### Contact with bats

Bat exposures were relatively common, with 15.5% (127/821) of respondents across all age groups indicating that they had previously handled a bat. Overall, 22% (48/218) of rural respondents had handled bats compared with 13% (78/597) of urban respondents (*χ*^2^ = 9.8, p = 0.0018) Twenty one percent of male respondents (63/304) had handled bats compared with 12% (64/517) of female respondents (*χ*^2^ = 10.2, p = 0.0014) (Table 1). Two respondents indicated that they had been bitten or scratched, but neither had sought PEP. The one respondent who was a bat handler by profession reported no bites or scratches. One respondent indicated that a family member had been scratched by the bat and PEP had been sought.

Follow-up interviews with 49 respondents that indicated prior bat exposure on the initial interview indicated that 31% (15/49) had handled bats in the five years prior to the second interview, and 69% (34/49) had handled bats more than five years before. While the majority of respondents (71%, 35/49) indicated that they only had a single bat exposure, almost one third of respondents (14/49) had multiple exposures. Respondents with bat colonies on their properties commented that they often had multiple exposures to bats in a single year.

### Reported reasons for handling bats

Respondents reported a range of reasons for handling bats (Table 2). The most commonly provided reason (29%, 14/49) was that the bat was either injured or sick. Eighteen percent (9/49) of exposures had occurred during bat education events or visits to wildlife zoos. Interestingly, two respondents reported that they had handled bats in Bali, Indonesia, a common holiday destination for Australian travellers. Sixteen percent (8/49) of this group reported encountering bats in their houses or sheds, while 12% (6/49)reported that the bats were either carried into their houses, or injured, by domestic pets (cats and dogs). ‘Other exposures’ included riding into a bat on a bicycle and being given an orphaned baby bat to hold by a professional bat carer.

**Table 2:** Reported reasons given by respondents for bat exposure (n = 49)

	**Respondents reporting bat exposures**
	% (n)*
Domestic pet attacked/retrieved bat	12 (6)
Bat inside house/shed	16 (8)
Injured or sick bat	29 (14)
Bat trapped in netting/fence	12 (6)
Exposure occurred during bat education event or visit to wildlife zoo (within Australia)	18 (9)
Exposure occurred during visit to wildlife zoo or temple (Bali, Indonesia)	4 (2)
Other exposure	10 (5)

*Percentages do not add up to 100% as some respondents had multiple exposures.

### Respondents reported knowledge of bat warnings

Overall, 42% (345/821) of respondents reported previously having seen or heard a warning about bats; with 48% (166/345) reporting seeing the warning on television. Approximately half (51%, 176/345) reported that the reason given for the warning was the risk of infection from contact with bats. Thirty seven per cent (126/345) could not recall what advice was contained in the warning, while 35% (121/345) said that the message was not to handle bats (Table 3).

**Table 3:** Responses by respondents to questions about ABLV warnings

	**All respondents**	**Previously handled bats**	**Have not handled bats**
	**% (n)**	**% (n)**	**% (n)**
Seen or heard warning about bats	42.0 (345/821)	55.1 (70)	39.6 (275)
Reported seeing the warning on television	48.1 (166/345)	34.3 (24)	51.6 (142)
Reported that the reason for the warning was bat’s ability to cause disease/infection	51.0 (176/345)	57.1 (40)	49.5 (136)
Could not recall the content of the warning	36.5 (126/345)	n/a	n/a
Recalled that the primary message was that they should not handle bats	35.1 (121/345)	38.6 (27)	34.2 (94)

Percentages can sum to greater than 100% as respondents were allowed multiple responses.

### Practices upon encountering an injured or trapped bat

If faced with an injured or trapped bat, the majority of respondents (73%; 600/821) reported that they would not handle the bat and that they would phone an animal welfare organization, ignore/avoid contact with the bat or euthanize it at a distance (Table 4 refers to responses not respondents). However, a quarter of respondents (25%; 206/821) indicated that they would handle the bat. Of the 206 respondents who indicated that they would handle the injured or trapped bat, 17% (36/206) stated that they would use their bare hands. Of those that stated they would not use their bare hands, 58% (95/165) said they would use any glove or readily available hand covering (including blankets and towels), while only 8.5% (14/165) indicated that they would use “thick, industrial gloves”. Among those who had previously handled bats, 32% (41/127) indicated that they would again handle a trapped or injured bat compared with 24% (165/694) of those who had never previously handled a bat (*χ*^2^ = 4.13, p = 0.0421). Of those respondents who reported previously hearing a warning about bats, 24% (83/345) stated that they would handle a trapped or injured bat. Surprisingly, 22% (27/121) of respondents who said that the main communicated message relating to ABLV was “not to handle bats” stated that they would handle a trapped or injured bat if they encountered one.

**Table 4:** Reported practices of respondents upon seeing a sick or injured bat

		**All respondents**		**Previously handled bats**		**Have not handled bats**		**Reported hearing a warning about bats**		**Reported hearing message “Not to handle bats”**	
		**%**	**(n/821)**	**%**	**(n/127)**	**%**	**(n/694)**	**%**	**(n/345)**	**%**	**(n/121)**
Handle bat	Handle and take to the vet/animal welfare organization	17.1	140	17.3	22	17.0	118	15.1	52	16.4	19
	Handle and take home to care	3.3	27	5.5	7	2.9	20	3.5	12	1.7	2
	Release it	2.2	18	5.5	7	1.6	11	2.9	10	2.6	3
	Cover it with a towel or net	2.1	17	3.2	4	1.9	13	2.3	8	3.5	4
	Put it in a box	1.3	11	2.4	3	1.2	8	0.9	3	1.7	2
	Euthanize up close: involves touching bat	0.9	7	2.4	3	0.6	4	1.5	5	0.9	1
Not handle bat	Phone an animal welfare organisation	54.9	451	49.6	63	55.9	388	55.1	190	56.0	65
	Ignore it/leave it alone	15.7	129	9.5	12	16.9	117	19.1	66	20.7	24
	Euthanize at distance: no touching	5.4	44	15.0	19	36.0	25	8.4	29	11.2	13

Percentages can sum to greater than 100% as respondents were allowed multiple responses.

### Practices on receiving a minor scratch or major scratch or bite

Respondents were asked what actions they would take if they received a minor scratch (one without blood) and a major scratch or bite (one with blood) (Table 5). For minor scratches, 14% (117/821) of respondents indicated that they would ignore the scratch, while 10% (85/821) reported they would wash the wound with water and 21% (171/821) would use an antiseptic. Overall, 38% (311/821) said they would immediately seek medical care. For major scratches or bites, 0.5% (4/821) of respondents indicated that they would ignore the scratch, 10% (80/821) reported they would wash the wound with water and 12% (95/821) indicated that they would wash the wound with antiseptic. In total, 74% (603/821) said they would immediately seek medical care. Participants who had previously not handled bats (75%; 521/694) were more likely to immediately seek medical care than those who indicated that they had previously handled bats (65%; 82/127) (p value = 0.0137). Those who reported hearing warnings about bats also commonly reported that they would immediately seek medical care (80%; 275/345).

**Table 5:** Reported practices of respondents if they received a minor scratch or a major scratch/bite

	**All respondents**		**Previously handled**		**Have not handled bats**		**Knowledge of ABLV**		**Message of not handle bats**	
	**Minor scratch**	**Major scratch or bite**	**Minor scratch**	**Major scratch or bite**	**Minor scratch**	**Major scratch or bite**	**Minor scratch**	**Major scratch or bite**	**Minor scratch**	**Major scratch or bite**
	**% (n)**	**% (n)**	**% (n)**	**% (n)**	**% (n)**	**% (n)**	**% (n)**	**% (n)**	**% (n)**	**% (n)**
Immediately seek medical care (emergency department or general practitioner)	37.9 (311)	73.5 (603)	27.6 (35)	64.6 (82)	39.8 (276)	75.1 (521)	47.3 (163)	79.7 (275)	56.0 (65)	81.9 (95)
Wash the wound with antiseptic	20.8 (171)	11.6 (95)	29.9 (38)	18.1 (23)	19.2 (133)	10.4 (72)	20.9 (72)	11.6 (40)	15.5 (18)	13.8 (16)
Make a routine appointment to seek medical care	20.0 (164)	15.6 (128)	17.3 (22)	18.9 (24)	20.5 (142)	15.0 (104)	19.7 (68)	12.5 (43)	15.5 (18)	11.2 (13)
Ignore	14.3 (117)	0.5 (4)	19.7 (25)	0.8 (1)	13.3 (92)	0.4 (3)	7.8 (27)	0.3 (1)	5.2 (6)	0 (0)
Wash the wound with water	10.4 (85)	9.7 (80)	15.8 (20)	16.5 (21)	9.4 (65)	8.5 (59)	8.7 (30)	9.3 (32)	11.2 (13)	10.3 (12)
Call a doctor or medical help-line for advice	3.5 (29)	3.7 (30)	3.9 (5)	3.2 (4)	3.5 (24)	3.8 (26)	3.5 (12)	3.5 (12)	6.0 (7)	2.6 (3)
Kill the bat	0 (0)	0.2 (2)	0 (0)	0.8 (1)	0 (0)	0.1 (1)	0 (0)	0.3 (1)	0 (0)	0 (0)
Keep the bat for testing	0 (0)	0 (0)	0 (0)	0 (0)	0 (0)	0 (0)	0 (0)	0 (0)	0 (0)	0 (0)

Percentages can sum to greater than 100% as respondents were allowed multiple responses.

## Discussion

Within the genus *Lyssavirus* twelve species and two phylogroups have currently been recognised, with rabies virus being the type species (serotype 1) [14]. While large numbers of serotype 1 (rabies) infections in humans have followed contact with vampire bats [22,23], only extremely small numbers of human lyssavirus cases globally have been linked to other bat species [4]. The low number of known lyssavirus infections in Europe, two each due to European bat lyssavirus-1 (EBLV-1) and European bat lyssavirus-2 (EBLV-2) which resulted in two fatalities [24-26], have been attributed to the secluded nature of those bats associated with EBLV and hence, minimal human-bat contact. In Australia, bats are highly mobile and frequently found in large camps close to, or located within, urban areas, making bat-human interactions a relatively common event, with the potential for ABLV transmission.

In our study, bat handling was relatively commonly (15.5%) reported. Two people reported bites or scratches and did not seek post exposure treatment (PET) nor notify public health authorities, the source of PET. In Brisbane, Queensland, while notifications requiring PET were higher immediately after the discovery of ABLV, with 189 notifications in the period November 1996 to October 2000, only 98 potential exposures were documented during each of the following four year periods [17].

Thirty one percent of exposures occurred in the five years prior to interview. Respondents with bat colonies located on their properties commonly reported multiple exposures. While 18% of exposures occurred during bat education events or visits to Australian wildlife zoos, these practices have been discontinued. However, there continues to be a risk of potential exposure to bat borne viruses in travellers to Bali, Indonesia, where bat handling is possible at bat temples and other tourist locations. The reported exposure of an unvaccinated and un-gloved respondent given an orphaned baby bat to hold by a professional bat carer is particularly disturbing as it occurred in 2013.

Domestic pets were involved in 12% of exposures, highlighting the potential for lyssavirus exposure in domestic pets. Infection from European Bat Lyssavirus 1 has previously been described in cats and sheep in Europe [27,28], both of which are dead-end hosts for rabies. McColl (2007) reported that, in a laboratory setting, cats and dogs seroconverted after ABLV exposure with some abnormal clinical signs in both cats and dogs. The results were inconclusive as to whether ABLV could then be further transmitted to humans or other animals [29].

A number of reported exposures (29%) were with obviously sick or injured bats. With ABLV being identified in up to 20% of sick, injured or orphaned bats, the most likely bats to be physically encountered by humans, it is perhaps surprising that more cases of ABLV have not occurred. With only three ABLV cases identified, the possibility remains that infections may go unrecognised by the attending clinician. ABLV is among a number of recently emerged pathogens in Australia which have presented with an encephalitis syndrome [30]. Up to 70% of adult encephalitis hospitalisations in Australia have no cause identified suggesting unrecognised or unidentified aetiologies [31]. The majority of these encephalitis cases undergo limited testing to determine causality [32]. A retrospective study in the Northern Territory found, amongst unexplained encephalitis cases, that very few samples were available for lyssavirus testing [33]. Improved encephalitis surveillance and the use of a standardised encephalitis testing algorithm, which includes ABLV, may help to address this issue.

The reported likely behaviours on encountering an injured or trapped bat would place survey participants at potential risk of ABLV exposure. A quarter of respondents reported that they would handle an injured or trapped bat and 17% would use their bare hands. Surprisingly, of those with knowledge of bat handling warnings (n = 121), a small number (n = 27) still stated that they would handle a trapped or injured bat.

Those ignoring a minor or major scratch could experience a fatal ABLV infection. Any contact with a bat is considered by the World Health Organization to be a category III exposure requiring PEP [14]. Current Australian clinical guidelines recommend that the bite or scratch site should be washed with soap and water and an iodine solution applied to the wound [16]. The National Health and Medical Research Council also recommend that:

“Post-exposure treatment should be considered whenever a bite, scratch or mucous membrane exposure to saliva from any Australian bat has occurred, regardless of the extent of the bite or scratch, the time lapsed since the exposure, the species of bat involved, and even if the bat was apparently normal in appearance and behaviour” [16].

The Health Belief Model [34] and the Protection-Motivation Theory [35] suggest that if a threat is perceived as severe, and there is a possibility that the event may occur to the individual, plus there is an action that the individual can take which will mitigate that risk, then a change in behaviour is likely to occur. The individuals also need to believe that the alternative, non-contact with bats, is acceptable. Based on study findings, risk communication has not adequately penetrated general community awareness. Future communication efforts need to adequately emphasise threat severity, the necessity of avoidance and promote the alternative of contacting trained bat handlers to manage trapped or injured bats and flying foxes.

As the study was conducted by telephone, those people without landlines or who were not connected to a telephone network were excluded. Although calls were attempted until 8 pm, the majority of calls were made during working hours, which may explain the overrepresentation of older persons and females. However, this bias would favour an under representation of bat exposures as our study indicates that males are more likely to handle bats than females. Responses to queries on likely behaviour on contact with sick or injured bats may not necessarily be indicative of actual behaviour. Recall bias may have affected self-reported ‘handling of bats’. The findings may not be representative of the total Australian population; in particular Queensland residents where the three ABLV cases have occurred.

## Conclusions

In our study, previous human-bat interactions were found to be relatively common. Potentially high risk practices were reported if respondents encountered an injured or sick bat, indicating a considerable potential risk for preventable ABLV exposures. The success of current messages advising against handling bats appears limited and new strategies must be considered particularly given the recent death in a Queensland child [11].

## Competing interests

The authors declare that they have no competing interests.

## Authors’ contributions

DD, MB, BP, KE, AJ and PM conceived the study. BP, MB and DD drafted the manuscript. BP, KE and MB interviewed participants. MB undertook the statistical analysis. All the authors read and approved the final manuscript.

## Pre-publication history

The pre-publication history for this paper can be accessed here:

http://www.biomedcentral.com/1471-2458/14/58/prepub
